# Impact damping and vibration attenuation in nematic liquid crystal elastomers

**DOI:** 10.1038/s41467-021-27012-1

**Published:** 2021-11-18

**Authors:** Mohand O. Saed, Waiel Elmadih, Andrew Terentjev, Dimitrios Chronopoulos, David Williamson, Eugene M. Terentjev

**Affiliations:** 1 Cavendish Laboratory, University of Cambridge, J.J. Thomson Avenue, Cambridge, CB3 0HE UK; 2 Cambridge Smart Plastics Ltd, 18 Hurrell Rd, Cambridge, CB4 3RH UK; 3 Institute for Aerospace Technology, University of Nottingham, Nottingham, NG7 2RD UK; 4 Department of Mechanical Engineering (LMSD), KU Leuven, Ghent Technology Campus, 9000 Gent, Belgium

**Keywords:** Mechanical engineering, Mechanical properties, Polymers

## Abstract

Nematic liquid crystal elastomers (LCE) exhibit unique mechanical properties, placing them in a category distinct from other viscoelastic systems. One of their most celebrated properties is the ‘soft elasticity’, leading to a wide plateau of low, nearly-constant stress upon stretching, a characteristically slow stress relaxation, enhanced surface adhesion, and other remarkable effects. The dynamic soft response of LCE to shear deformations leads to the extremely large loss behaviour with the loss factor *tan*δ approaching unity over a wide temperature and frequency ranges, with clear implications for damping applications. Here we investigate this effect of anomalous damping, optimising the impact and vibration geometries to reach the greatest benefits in vibration isolation and impact damping by accessing internal shear deformation modes. We compare impact energy dissipation in shaped samples and projectiles, with elastic wave transmission and resonance, finding a good correlation between the results of such diverse tests. By comparing with ordinary elastomers used for industrial damping, we demonstrate that the nematic LCE is an exceptional damping material and propose directions that should be explored for further improvements in practical damping applications.

## Abstract (web summary)

Nematic liquid crystal elastomers (LCE). exhibit unique mechanical properties such as large loss behaviour, which makes these materials interesting for damping applications. Here, the authors investigate the effect of anomalous damping in LCEs by comparing impact dissipation in shaped samples with elastic wave transmission and resonance.

## Introduction

There is an increasing interest in new materials that can efficiently dissipate mechanical energy, both from a fundamental understanding of viscoelasticity and practically in the area of suppressing vibration and protecting from impact. Examples of applications with high demand in damping include the automotive, aerospace, wind energy and home appliance industries, but in reality, the list is much wider. The damping of vibration energy arises due to the energy loss processes in a viscoelastic material that convert the vibrational energy into heat in either free or constrained layer configurations^1,2^. The characteristics of the damping material are reflected in the linear complex modulus E*(*f*) at a given oscillation frequency *f*, characterised by its real and imaginary parts: the storage and loss moduli *E*′ and *E*″, and their ratio *E*″/*E*′, usually referred to as the loss factor tan *δ* with *δ* the phase angle of E*^3^. The loss factor tan *δ* is directly related to the other parameters describing damping: tan *δ* = 2*ζ* = 1/*Q*, where the ‘quality factor’ *Q* and the ‘damping ratio’ *ζ* are commonly used in the analysis of resonance circuits and engineering constructions. In mechanical engineering, it is known that merely by assembling a metal construction with joints and fasteners, already produces a resonance damping with *Q* < 100 (tan *δ* > 0.01), so a substantial increase above this is desired. In addition, for optimal damping, it is desirable to have a high loss factor over a wide temperature and frequency ranges. With most current polymeric materials for damping, the properties are optimised only for particular values of temperature and frequency, often corresponding to their glass transition, where the tan *δ* has a large peak^2,4^. The ideal materials must also have other attributes to be effective in practical situations, such as thermal stability, ease of forming complex shapes, good adhesion, possibility for large-scale manufacture, and ease of application.

Liquid crystalline elastomers (LCEs) are amorphous rubbers with spontaneous orientational order of their anisotropic molecular segments. They possess the combination of physical properties that place them in a separate category from any other elastic or viscous material^5^. The nature of this uniqueness lies in coupling of rubber elasticity and liquid crystalline degrees of freedom in the elastomer matrix. In ordinary elastic solids, the deformations are created by the relative movement of the same atoms (or molecules) that form the bonded low-symmetry lattice. Hence, when the deformation is small, the lattice symmetry is preserved, and one obtains an ordinary elastic response (although sometimes anisotropic); large deformations destroy the lattice integrity and break the material. In elastomers and gels, the macroscopic elastic response arises from the entropy change of polymer chains on stretching their crosslinked end points, which are far apart. What happens to chain segments on a smaller length scale is a matter relatively independent of what defines rubber elasticity. For instance, when the orientational nematic order is established within network strands, its director could rotate independently from the deformation of crosslinking points. Such an internal orientational degree of freedom within, and coupled to the elastic body, constitutes what is known as the Cosserat medium^5,6^, an example of an elastic medium with a mobile internal microstructure^7^. However, the LCEs are even richer than a nominal Cosserat solid since rubbers are capable of large shear deformations, being at the same time essentially incompressible. Hence, one expects, and indeed finds, a variety of unique mechanical properties described in some detail in the defining book on the subject^5^.

The striking feature of LCE having been well studied in the equilibrium regime is the ‘soft elasticity’, which manifests as a wide, almost zero-stiffness plateau of low nearly constant stress upon increasing strain, and is caused by internal rotation of the local director axis, which absorbs the applied strain without an elastic energy cost^8–10^. The interest in ‘dynamic soft elasticity’ of nematic LCEs^11–14^, has been driven by the unusual aspects of the dynamic-mechanical response, and in particular, the unusual properties of elastic waves propagating in LCE^15,16^. One of the key features is the anomalous mechanical damping, increasing with the increased vibration frequency^16^. Notably, only the nematic phase of LCE was proven attractive for vibration damping: in smectic LCEs^17^, the coupling between crosslinks and the layer positions leads to strong internal constraints resulting in the pronounced hysteretic shape-memory effects^18,19^.

Here we study and compare the impact damping and vibration attenuation of nematic LCE materials, optimising the geometry of impact or vibration deformation to maximise the internal shear component that initiates the anomalous LCE dissipation. We also optimise the choice of the nematic LCE, taking into account the required energy damping maximisation and the practicalities of industrial material production. We also compare a range of LCE against current market-leading damping materials, using different testing methods for impact absorption and vibration (elastic) attenuation.

## Results

### Characterisation of LCE materials

**Fig. 1:**
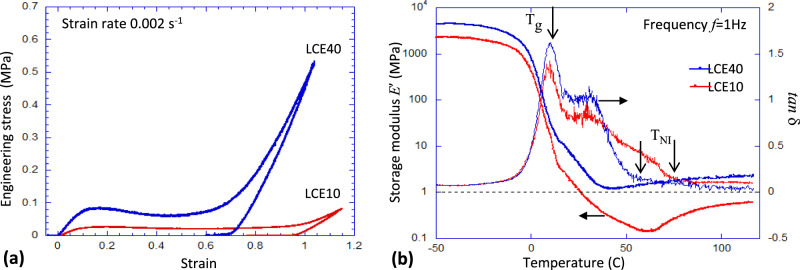
Mechanical characterisation of LCE materials. **a** Near-equilibrium stress–strain curves for LCE10 and LCE40 materials, highlighting the wide soft plateau through internal re-orientation of the polydomain LCE on stretching, as well as the ability to withstand large deformations. **b** Temperature scan of linear oscillating response (at fixed 1 Hz), highlighting the high tan *δ* across all of the nematic range.

The best current LCE materials are based on the robust and error-proof ‘click’ chemistry of thiol-acrylate Michael addition^20^, using the cheap commercially available starting materials and easily controlled crosslinking density (Supplementary Fig. S1). For practical applications, we need naturally non-aligned, polydomain LCEs^21^ with relatively low crosslinking density, which show the wide stress plateau reflecting the elastic softness on nematic director alignment (see Fig. 1a), comparing the basic LCEs with two crosslinking densities: 10% and 40% (as labelled in the plot). Figure 1b shows the typical oscillating dynamic-mechanical response of an LCE, scanning the temperature range at a fixed frequency, again comparing the crosslinking densities in the same materials labelled LCE10 and LCE40, respectively. The result illustrates the key regimes of the dynamic response: the rigid low-dissipation glass below a glass transition *T*_g_, the low-modulus high-damping nematic range below the nematic-isotropic transition *T*_NI_, and the ordinary isotropic rubber above *T*_NI_. The anomalous damping window between the glass at low temperature and the isotropic phase at high temperature is roughly [5–60 °C] in these LCEs. There is a large literature on how one can control both these key transitions by chemical modifications, moving the transition temperatures up or down (see the discussion in Supplementary); this was not our focus in this study. While the test in Fig. 1b shows the components of complex tensile modulus E*(*ω,T*), the theory of LCE response^11,12^ makes it clear that only the shear deformation carries the anomalous dynamic soft elasticity; not surprisingly, the early published data showed much higher tan *δ* values, being tested in the pure shear geometry^10,14^.

**Fig. 2:**
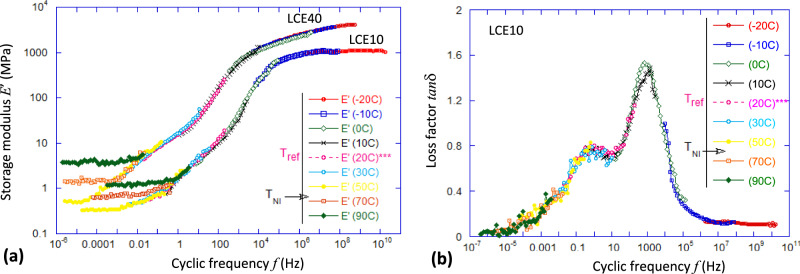
Master Curves of LCE materials. **a** The tensile storage modulus *E*′(*ω*) for LCE10 and LCE40 materials, obtained by time–temperature superposition of frequency-scan tests at different temperatures (labelled in the plot) with the frequency scaled for the reference *T* = 20 °C. The nematic transition *T*_NI_ for both materials is between 50 and 70 °C and indicates the end of dynamic softness (the isotropic rubber modulus increases on heating). **b** The corresponding Master Curve of the loss factor tan *δ* for LCE10, with the frequency scaled for the same reference *T* = 20 °C. Unlike the storage modulus *E*′, whose magnitude at high temperatures is affected by the nematic-isotropic phase transition and the entropic rubber elasticity, the time–temperature superposition for the loss factor works well at this high-temperature/low-frequency region.

Figure 2a presents the Master Curves of frequency dependence of the linear dynamic modulus (tensile modulus, as in Fig. 1b). Note that above the glass transition one has *E* ≈ 3 G, given the effective incompressibility of rubbers and the average isotropy of polydomain LCE, with the bulk modulus remaining high: *K* > 2 GPa. These Master Curves have the frequency scaled by time–temperature superposition^11,12^, using the *T* = 20°C as the reference temperature; the unusual behaviour of *E*′(*ω*) at low frequencies reflects the dynamic soft elasticity when the quasi-equilibrium modulus in the nematic LCE phase is much lower than in the isotropic phase above *T*_NI_. Unlike in ordinary polymers, where the Williams–Landel–Ferry (WLF) time–temperature superposition^3^ is based on the changing timescales during the glass transition, in nematic LCE (well below its *T*_g_) the rubbery response adds the additional complexity: the lower modulus in the nematic phase due to the internal director relaxation modes (the origin of anomalous damping), and the increasing rubber modulus in the isotropic phase above *T*_NI_ (due to the entropic rubber elasticity *E* ~ *k*_B_*T*). Supplementary Fig. S2 gives more detail on this procedure and the building of Master Curves in LCE.

Figure 2b shows the same time–temperature superposition for the dynamic loss factor tan *δ*, obtained by scaling the frequency of the tests carried out at different temperatures (as labelled in the plot, in the same format as in Fig. 2a). Here, unlike for the storage modulus in Fig. 2a, the frequency scaling works well for high temperatures, producing the predicted values at ultra-low frequencies (for the reference temperature of 20 °C). The frequency band where LCE materials show high damping is roughly between 0.1 Hz and 20 kHz at ambient temperature. As usual in time–temperature superposition, this window proportionally shifts to higher frequencies for a higher reference temperature, and vice-versa for a lower reference temperature. However, one should be careful with interpretation, since the nematic–isotropic phase transformation occurs around *T*_NI_ = 60 °C in these LCE materials: if the actual LCE is tested above 70 °C, it will be plain isotropic and its vibration damping will not be different from ordinary elastomers irrespective of the testing frequency. This is because the WLF time–temperature superposition is based on the (empirically validated) assumption that the internal dynamics are controlled by the ‘cage confinement’ in the glass state, which gradually gets weaker and eventually gets fully released in the melt state. In the nematic LCE, the internal dynamics are slowed down by the orientational relaxation modes, but only at a temperature within the thermodynamic nematic phase: unlike glass, a different thermodynamic phase cannot be produced by changing the input frequency of probing oscillation.

### Testing of impact damping

The impact damping was tested in the Hopkinson split pressure bar experiment and also in a separate but closely related ball-impact test. The Hopkinson bar test is based on the impact of two parallel faces of metal bars, with the test sample inserted in the impact area; it is well described in the ASM Handbook volume^22^. However, we quickly established that inserting a flat elastomeric pad in such an impact only activates the compressional deformation modes, and therefore shows no significant effect of the LCE dynamic softness: the test probes the bulk modulus, which is approximately the same in all elastomers (see Supplementary for further detail and plotted data). LCE10, LCE40, as well as ordinary siloxane and thermoplastic polyurethane elastomers from the market-leading vibration damping suppliers Sylgard^®^ and Sorbothane^®^, respectively, all showed the same effect of dissipating about 25–27% of impact energy in this geometry.

**Fig. 3:**
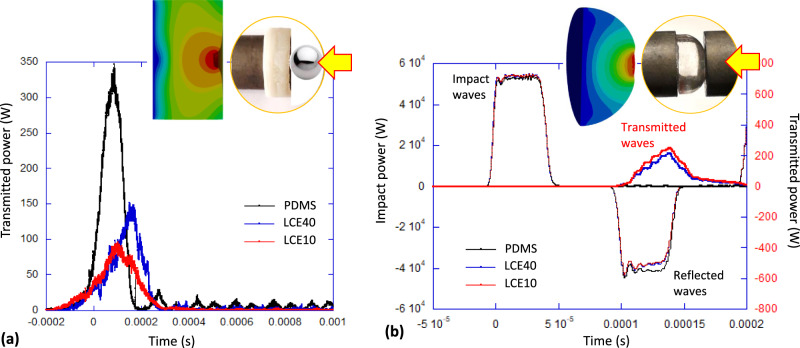
Impact damping test. **a** Spherical ball impact on a flat elastomer pad. The incident kinetic energy of ca. 3 J was partially transmitted through the elastomer into the receiving bar, the plot showing the power transmitted after the impact at *t* = 0 s. PDMS sample has 74% of impact energy dissipated, and clearly shows the under-damped oscillations after impact. Both LCE samples have dissipated over 90% of impact energy, and show the overdamped response both at the onset and after the force pulse. **b** Flat surface impact on a semi-spherical elastomer pad (of PDMS, LCE40 and LCE10), showing the low and heavily overdamped wave transmission: of the impact energy of 2 J, only 0.01 J (0.4%) was transmitted into the target rod by the LCEs while almost all energy was reflected by PDMS, with 28% of impact energy absorbed in the elastomers in this geometry. In both panels, the sketches illustrate the geometry of impact and the deformation field distribution in the elastomer.

For this reason, we looked into exploring the impact geometry where the shear deformation modes are excited, the most straightforward being the impact of a flat elastomeric pad with a spherical projectile ball, which initially produces spherical waves from the point of impact. This was tested in two settings: Fig. 3a shows the results of impacting a flat elastomer pad with a spherical projectile, while Fig. 3b presents the impact of the flat face of the Hopkinson split pressure bar on a semi-spherical cap of the elastomer. In both cases, a significant shear deformation component is generated in the material, and the comparison between the ordinary elastomers and LCE is stark. Integrating the measured power over time allows us to estimate the full energy budget: the initial kinetic energy converting into the transmitted, reflected, and dissipated “lost” energy. For example, for the spherical projectile impact on an elastomer pad of 3 mm thick, with the initial energy of 3 J, we found that the PDMS had 25% of energy in the rebound, 1.2% of energy transmitted, with ca. 74% of impact energy dissipated. For the same 3 mm-thick LCE40 pad the results showed 6% of energy in the rebound, 0.7% of energy transmitted, with ca. 93% of impact energy dissipated, and for LCE10: 5% of energy in the rebound, 0.6% of energy transmitted, with ca. 94% of impact energy dissipated.

In the impact of the flat face of split Hopkinson bar on a semi-spherical cap of an elastomer, of 10 mm diameter (Fig. 3b), receiving a strike with the impact energy of 2 J, we found that the PDMS cap had almost no energy transmitted, but rebounded 79% of impact energy (with 21% energy loss). The LCE damping caps did transmit a very small fraction of impact energy: LCE40 transmitted 0.3%, rebound 72% (loss 27.5%), and LCE10 transmitted 0.4%, rebound 71% (loss 28.5% of impact energy). It is clear that the shape and dimensions of the damping pad play a significant role in this energy distribution. However, we note that even with spherical-cap shaped pads, the mechanical loss in the material was comparable between PDMS and LCE systems, and much lower than in the projectile-impact test (where very little energy was in the rebound). In both tests, the PDMS elastomer had a distinct elastic response with resonance bounces, in contrast to both LCE materials showing a strongly overdamped response.

It is important to establish a correspondence between the impact measurements, providing data in real time, and the frequency domain where we see both the material properties (such as Master Curves in Fig. 2) and the elastic waves. For this, we examined the spectral distribution of power transmission in the ball impact, (Fig. 3a, also see Supplementary Fig. S3). As expected for the sharp, 0.2 ms pulse in real time, its frequency distribution is a relatively flat-top until the cutoff frequency for PDMA at ca. 8 kHz, and ca. 10 kHz for both LCE40 and LCE10. This frequency range captures almost all of the enhanced-tan *δ* region in the Master Curve in Fig. 2(b), which explains the almost complete dissipation of the impact energy in the material.

### Testing of vibration attenuation

**Fig. 4:**
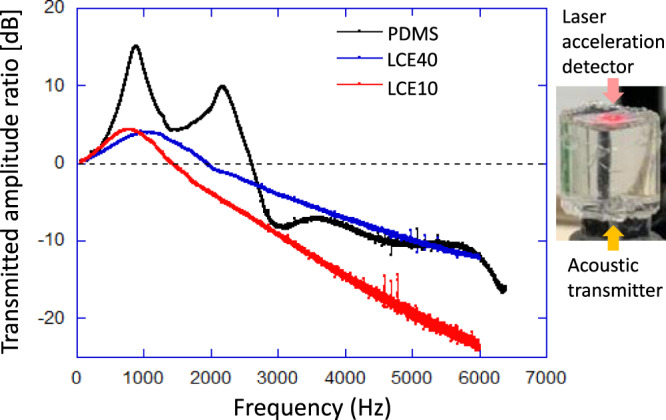
Vibration attenuation test. The amplitude of the signal (measured in [dB] as a ratio to the incident amplitude) transmitted through the elastomer cylinder is plotted against the vibration frequency. The primary standing wave resonance near 1 kHz is found in all materials, with underdamped PDMS showing several secondary resonance peaks. The overdamped LCE systems have the *Q*-factor equal to 0.4 (i.e. the damping ratio *ζ* = 120%), compared with PDMS: *Q* = 2.4 (*ζ* = 21%). Supplementary Fig. S4 shows graphical representations of resonance modes.

The study of transmission and attenuation of vibrations, particularly in the sonic range from 50 to 6000 Hz, were carried out on a home-made device, described in Supplementary Fig. S6. To generate spherical waves^23^, and thus explore their shear deformation component, the cylindrical elastomer sample was held upright; this way, the elastic waves were initially radiating spherically before the resonant standing wave pattern was established in the cylindrical samples. Three sets of cylindrical samples were prepared with a similar length of 15–16 mm and a cross-sectional diameter of 17 mm (by crosslinking in the mould). Two samples were LCE 10 and LCE 40, and one sample was PDMS, used mainly for comparison and benchmarking. Each sample was mounted on a dynamic shaker that sends elastic waves longitudinally through the bottom surface of the sample, as illustrated in Fig. 4. A dynamic laser interferometer was used to pick up the acceleration at the top surface (the output signal from the structure), and an accelerometer was used to measure the acceleration at the bottom surface (the input signal to the structure).

Figure 4 shows the damped-oscillator wave resonance of the system, again comparing LCE materials with the standard PDMS elastomer. The results show resonances of the three samples at around 1000 Hz (see Supplementary Fig. S7 for detailed modal analyses). The resonance of the PDMS has a peak value of 15 dB. In reference to the PDMS resonance peak, the LCE samples showed a much lower peak response which was around 4.5 dB. These peak values of the LCE samples are more than two times less than that of the PDMS sample for similar size, form and vibration mode. This suggests the existence of higher damping values in the LCE samples in comparison to the PDMS.

In comparison to the results in the impact case shown earlier, a wide-area transducer generating longitudinal compression waves shows no effect of the LCE dynamic softness. In the impact case, the waves of compressional deformation propagate and attenuate similarly to all elastomers. We measured the speed of longitudinal elastic wave travelling along the cylinder length (ca. 15 mm), as *c* = 49 m/s for PDMS, 43 m/s for LCE10, and 62 m/s for LCE40. Given that the density of all elastomers is similar, ca. 1200 kg/m^3^, these values confirm that it is the shear (rubber) modulus that is responsible for the acoustic wave: the typical values of rubber modulus are *G* = 1–2 MPa (for the modulus at a given frequency one should consult the Master Curve in Fig. 2a), which closely matches the measured wave speed $c\approx \sqrt{G/\rho }$ (the precise value of wave speed is affected by many additional factors: from the sample shape to the elastic impedance). It is also consistent that LCE10 and PDMS had approximately the same crosslinking density (hence a similar rubber modulus), and therefore a comparable wave speed, while LCE40 had a higher crosslinking density, and accordingly, a slightly higher wave speed. For comparison, the bulk modulus of all elastomers is approximately the same, *K* = 1–2 GPa, predicting a typical speed of longitudinal compression wave in the order of 1000 m/s: such a fast wave zips across the 15 mm distance in ca. 15 μs, and therefore would affect the measurement above 60 kHz, well outside our testing range.

The attenuation of the acoustic waves in the elastomers was measured via the damping of the primary resonance peak, which was found around 1 kHz in all materials, consistently with the separately measured wave speed and the distance to travel. The quality factor *Q* of the resonance, defined as the frequency-to-bandwidth ratio of the resonator is calculated using the −3 dB method^24^, was determined as *Q* = 2.4 for PDMS (the damping ratio *ζ* = 21%, indicating the underdamped material). This is consistent with several secondary resonances seen in the PDMS transmission data in Fig. 4 and also with the secondary vibrations in the impact test seen in Fig. 3a. In contrast, the LCE materials are heavily overdamped: *Q* = 0.42 (*ζ* = 120% for LCE40, and *Q* = 0.45 (*ζ* = 110%) for LCE10. The difference between LCE10 and LCE40 is within the uncertainty of the test, influenced by precise details of the surface cut, the cylinder width and length, and even the placement of the transducer. It is consistent with the theoretical understanding that the anomalous vibration dissipation, on the microscopic level, is caused by the local rotations of the nematic director, which occur on the length scale well below the network mesh size given by crosslinking density (an extensive discussion of this theory can be found in refs. ^4,11,12^).

## Discussion

There are three aspects that we touch on in this paper: the choice of LCE material for optimised mechanical damping, the effect of the sample shape, and the comparison of damping tests done in the time domain (impact damping) and in the frequency domain (acoustic attenuation). The early damping studies were conducted on side-chain LCE^16^, and have demonstrated a higher loss factor tan *δ* than we see in Fig. 2b. However, the mechanical strength and ductility of side-chain LCE are notoriously low, and we have instead chosen to work with the new generation of main-chain thiol-acrylate LCE^20^, on which there have already been a number of recent investigations^25,26^. Although the loss factor seldom exceeds 0.8 in these materials, the mechanical strength and durability make them a good choice for many application settings. We have discussed in the text, and quoted the literature on elastic wave theory, to highlight the fact that to reach the desired regime of dynamic softness (and with it, the enhanced damping), one must ensure that shear deformation occurs in the material. The geometries that are sustained by compressional deformations are controlled by the high bulk modulus of elastomers and make a little damping effect. To achieve these shear deformations, we elected to generate spherical waves: radiating from an impact point of projectile or emitted by a small transducer, or reflected from a spherical sample boundary. Note that in the latter case, the amount of dissipated energy was much lower than when genuine spherical waves were generated (see the discussion of Fig. 3b).

Our main focus was on comparing and contrasting the two testing approaches: the impact study, which explores the process in real time, and the study of elastic wave propagation that looks for frequency-dependent transmission and resonances. The narrow pulse of impact is a sum of many frequencies, but even though we did examine its spectral distribution (Supplementary Fig. S5) is not easy to quantitatively relate the two techniques due to the different excited ranges and also the finite size and shape of the samples in both measurements. Two important correlations reassure us that they both return compatible results. The underdamped elastic response of PDMS shows in different ways in the two tests, but is clear and unambiguous—as is the heavily overdamped nature of LCE materials. The ‘slowing down’ of the response to impact means that the acceleration that the projectile experiences is lower (since the time of the impact going in and back out is longer). This has an important implication for the use of LCE as protective layer, because it is the acceleration of the impactor that determines its fate (see, for instance, the ASTM F2656 standard test method for crash testing of vehicles). Turning to the other side of the protective pad, we highlight the low amount of energy transmitted through even a thin pad into the target. We are not sure why there was no registered transmission in the case of a semi-spherical PDMS cap with a flat-face strike, but we suspect it was a fortuitous accident of internal resonance in the elastic medium, which resulted in the full rebound. Looking at all the results, it is clear that the impact transmission through the LCE protective pad is consistently very low, which invites a different set of applications (when the stationary object behind the pad is protected).

Our aim was to present and compare the two testing methods, impact and acoustic, and bring them closer to some standard that could be consistently applied in different applications and materials, as well as summarise the LCE characterisation via Master Curves. For advanced applications, this field will clearly develop into using composites, generating complex shear-rich local deformations in the matrix, with highly damping LCE dispersed in elastic structures: this is already an approach pursued in the recent literature^13,24^. The research field will extend towards including LCE materials within layered and latticed composite structures, resulting in constrained damping layer technologies of superior performance. Furthermore, LCEs have excellent properties for inclusion within low-frequency damping and sonic sealing applications.

## Methods

### Materials and preparation of LCE

For the preparation of LCE, we followed the methods reported previously, with a single-step crosslinking reaction of thiol-acrylate Michael addition^27^. The diacrylate monomer, 1,4-bis-[4-(3-acryloyloxypropypropyloxy) benzoyloxy]−2-methylbenzene (RM257) was purchased from Daken Chemical Co. The dithiol spacer, 2,2′-(ethylenedioxy) diethanethiol (EDDET), and tetrathiol crosslinker pentaerythritol tetrakis (3-mercaptopropionate) (PETMP), were purchased from Sigma Aldrich. Triethylamine (TEA, Sigma Aldrich) was used as the catalyst of the thiol-acrylate Michael-addition reaction. As the radical scavenger, butylated hydroxytoluene (BHT, from Sigma Aldrich) was used to suppress the unwanted radical polymerisation reaction between acrylates. All chemicals were used in their as-received condition with no purification.

At the specific molar ratio of functional groups shown in Supplementary Table S1, RM257, EDDET, PETMP and BHT (0.5 wt%) were weighed. After each mixture was gently mixed at an elevated *T* ~90 °C for ~10 min, TEA was added at 0.8 wt% to start the Michael-addition reaction between thiol and acrylate groups. The mixture was then transferred into a Teflon mould to complete the polymerisation at 90 °C (isotropic phase) overnight.

### Stress–strain response

The stress–strain curves for LCE films on the tensile mode were obtained using a home-made tensile testing rig, delivering a controlled deformation rate over a large range (to cover a large sample extension needed to pick all key regimes of LCE stress response). The measured tensile force was converted into engineering tensile stress by dividing over the initial sample ratio.

### Dynamic-mechanical characterisation

The small-amplitude oscillating measurements were carried out on TA DMA 850 instrument in tensile mode. The constant-frequency temperature scans were performed to identify the key phases of the materials (glass, nematic, isotropic rubber). For this, we used the low frequency of 1 Hz to obtain the storage modulus closer to the expected equilibrium values of Young’s modulus in different phases. The elevated values of the loss factor indicated enhanced mechanical dissipation across the nematic phase.

The time–temperature superposition was used to produce dynamic Master Curves of elastomers at a chosen reference temperature. As is standard in the WLF method, we carried out frequency scans in the available range of 0.01–200 Hz at different temperatures, and then scaled the frequency by a temperature-dependent factor *α*(*T*) to achieve the overlapping of consecutive scans. This was successful across the glass transition (for which the WLF method is designed); however, crossing the nematic–isotropic transition makes it impossible to overlap the modulus curves since the overall modulus magnitude changes (downwards) due to the nematic softness and (upwards) due to the entropic rubber-elastic effect on heating. We left the high-temperature data un-scaled across the nematic transition for the *E*′(*ω*) Master Curves. In contrast, the superposition of the loss factor worked perfectly across the nematic–isotropic transition.

### Split Hopkinson bar experiment

The classical home-made setup involves two identical bars (using soft magnesium to improve the impedance ratio between the metal and the elastomer), with the sample fixed on the front face of the “transmitted bar”, waiting to be struck by the “incident bar”, which is accelerated by the controlled pneumatic striker. The sequence of piezoelectric strain gauges is fixed on both bars to register the longitudinal elastic wave travelling along each of them. The overall energy balance of initial and reflected elastic wave energy in the incident bar, plus the wave energy in the transmitted bar, allows us to accurately find the amount of energy dissipated in the elastomer.

In a modification of this test, we have replaced the incident bar with an air gun firing a steel ball projectile, with a sequence of LVDT sensors registering its speed as the ball passes along the barrel initially, and after reflection. The strain gauge in the transmitted bar provides the data on transmitted force and velocity, which can be converted into the transmitted power. The overall energy balance of initial and reflected kinetic energy of the projectile, plus the mechanical energy transmitted into the bar, allows to find the dissipated energy.

### Elastic wave attenuation experiment

The acoustic attenuation was studied on a home-made laser vibrometer setup. A dynamic shaker (Modal Shop K2007E01) was used to generate a periodic chirp signal, generating a surface acceleration ranging between 0.2 and 0.6 m/s^2^. The dynamic force at the input surface of the structure was measured using an impedance head (PCB 288D01)with a sensitivity of 22.4 mV/N. The internal resonance of the setup comprising the impedance head and dynamic shaker occurred at 7500 Hz; thus our measurement was restricted to below 6000 Hz. The acceleration at the output surface of the structure was measured by a laser vibrometer system (Polytec PDV-100), mounted vertically above the flat cylinder surface. The ratio of output-to-input amplitude was converted to dB for presentation.

## Supplementary information

Supplementary Information

## Data Availability

Data that support the findings of this study are available from the corresponding author upon request.
