# Supplementary material for: Impact damping and vibration attenuation in nematic liquid crystal elastomers
Source: Nat Commun. 2021 Nov 18;12:6676. doi: 10.1038/s41467-021-27012-1 (PMC8602409; doi:10.1038/s41467-021-27012-1)
Supplement: Supplementary file 1 — Supplementary Information [file 41467_2021_27012_MOESM1_ESM.pdf]

Supplementary Information

Impact damping and vibration attenuation in nematic liquid crystal elastomers

Mohand O. Saed, Waiel Elmadih, Andrew Terentjev, Dimitrios Chronopoulos, David Williamson, Eugene M. Terentjev

1. Material preparation

Isotropic PDMS network: Using the Sylgard 184 silicone elastomer kit (Sigma Aldrich), we mixed 18 g of PDMS with 2g of the curing agent to fabricate crosslinked silicone elastomer (PDMS – 10). After rigorously mixing the PDMS and curing agent, the mixture was degassed and then transferred into a mould (e.g. rectangular, cylindrical, or spherical shapes) and then placed in an oven at 60C for 6 hours to fully cure.

LCE networks: LCE samples were synthesized via a thiol-acrylate Michael addition reaction. LCE networks were prepared starting with two thiol monomers. The thiol monomers were selected for their use as a *multi*-functional crosslinking monomer and *di*-functional flexible spacer between mesogens. The flexible spacer (EDDET) was mixed with *tetra*-functional PETMP. The ratio of thiol cross-linker to flexible spacer was systematically varied using 10, or 40 mol % of functional groups belonging to the cross-linker. At the specific molar ratio of functional groups shown in Table S1, RM257, EDDET, PETMP, and BHT (0.5wt %) were weighed in a stoichiometric balance. The mixture was gently mixed at an elevated T ~90°C for ~10 min. Once the solution returned to room temperature, 0.8 mol % of TEA was added to catalyze the reaction. The solution was mixed vigorously using a Vortex mixer (No: 94540, Toronto, ON, Canada). Air bubbles were removed from the solution under a 500 mmHg vacuum. The solution was then injected between two glass slides separated with 1 mm spacers or transfer into a Teflon mould (e.g. rectangular, cylindrical, or spherical shapes) to complete the polymerization at 80°C (in isotropic phase) overnight.

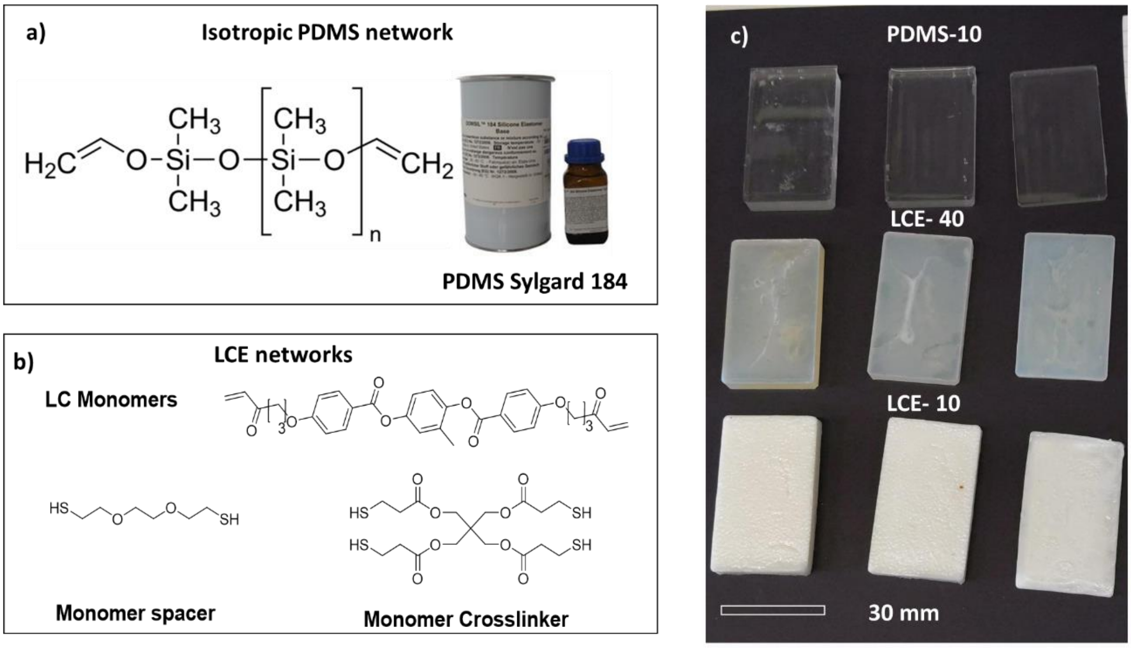

Fig. S1. Preparation of damping materials. (a) Isotropic PDMS network (b) LCE networks (c) pictures of the resulting materials.

The thermal transition temperatures of our LCEs are shown in the Table S1. The glass transition temperature (T_g) is measured at the temperature corresponding to the peak of the *tan*δ curve. T_g is 9 ˚C for LCE-10 and 10 ˚C for LCE-40. The nematic to isotropic transition temperature (T_{NI}) is measured at the point corresponding to the plateau onset of the *tan*δ. T_{NI} is 76˚C for LCE-10 and 68 ˚C for LCE-40. Previous studies have shown that the thermomechanical properties of the thiol-acrylate LCE systems are highly controllable. The thermomechanical properties of this system are directly linked to the structure and the amount of the starting monomer materials.[a] For example, T_g can be controlled through the structure and amount of the monomer crosslinker,[b] and T_{NI} can be controlled via the structure and amount of the monomer spacer.[c]

Table S1. Weight of the monomer used to prepare LCEs with 10% or 40% crosslinking concentration, measured in proportion to the fixed amount of RM257 monomer. The weight of catalysts is 1 wt% (0.27g).

	RM257 (g)	EDDT (g)	PETMP (g)	T_g	T_{NI}
LCE-10	20	5.7	0.85	9 ˚C	76 ˚C
LCE-40	20	3.8	3.4	10 ˚C	68 ˚C

a- Yakacki, C. M., et al. "Tailorable and programmable liquid-crystalline elastomers using a two-stage thiol–acrylate reaction." *RSC Advances* 5 (2015) 18997-19001.

b- Saed, M. O., et al. “Thiol-acrylate main-chain liquid-crystalline elastomers with tunable thermomechanical properties and actuation strain.” *J. Polym. Sci. Part B 55* (2017) 157-168.

c- Saed, M. O., et al. “High strain actuation liquid crystal elastomers via modulation of mesophase structure.” *Soft Matter, 13* (2017) 7537-7547.

2. Master Curves

The building of Master Curves, such as in Figure 2 in the main text, is done using the time-temperature superposition. It is important to draw a distinction between the classical WLF technique, which was designed to capture the glass transition in the polymer melt, and our case. First key distinction is that in a crosslinked thermoset (rubber), the low frequency high-temperature data cannot be superposed directly because of the linear T-dependent growth of the equilibrium rubber modulus. This requires an additional proportional scale-down of the storage modulus amplitude at temperatures above the reference point. Secondly, with the nematic phase below T_{NI}, there is an additional effect of dynamic softness (also at low frequencies), which reduces the storage modulus amplitude, and requires an adjusted scaling if the reference temperature is in the nematic phase. Overall, this is a delicate process that requires thought, and frequent comparison with the ‘reciprocal’ DMA curves of the T-dependence at a constant frequency. At the same time, we find that the superposition of *tan*δ(ω) is much more robust and does not require any additional amplitude re-scaling, so the frequency shift factors, $\omega' = \alpha(T)\omega$, are very systematic and can be used as a benchmark for the $E'(\omega)$ data. The example plots in Fig. S2 illustrate these points for the LCE10 material, leading to Figure 2 in the main text.

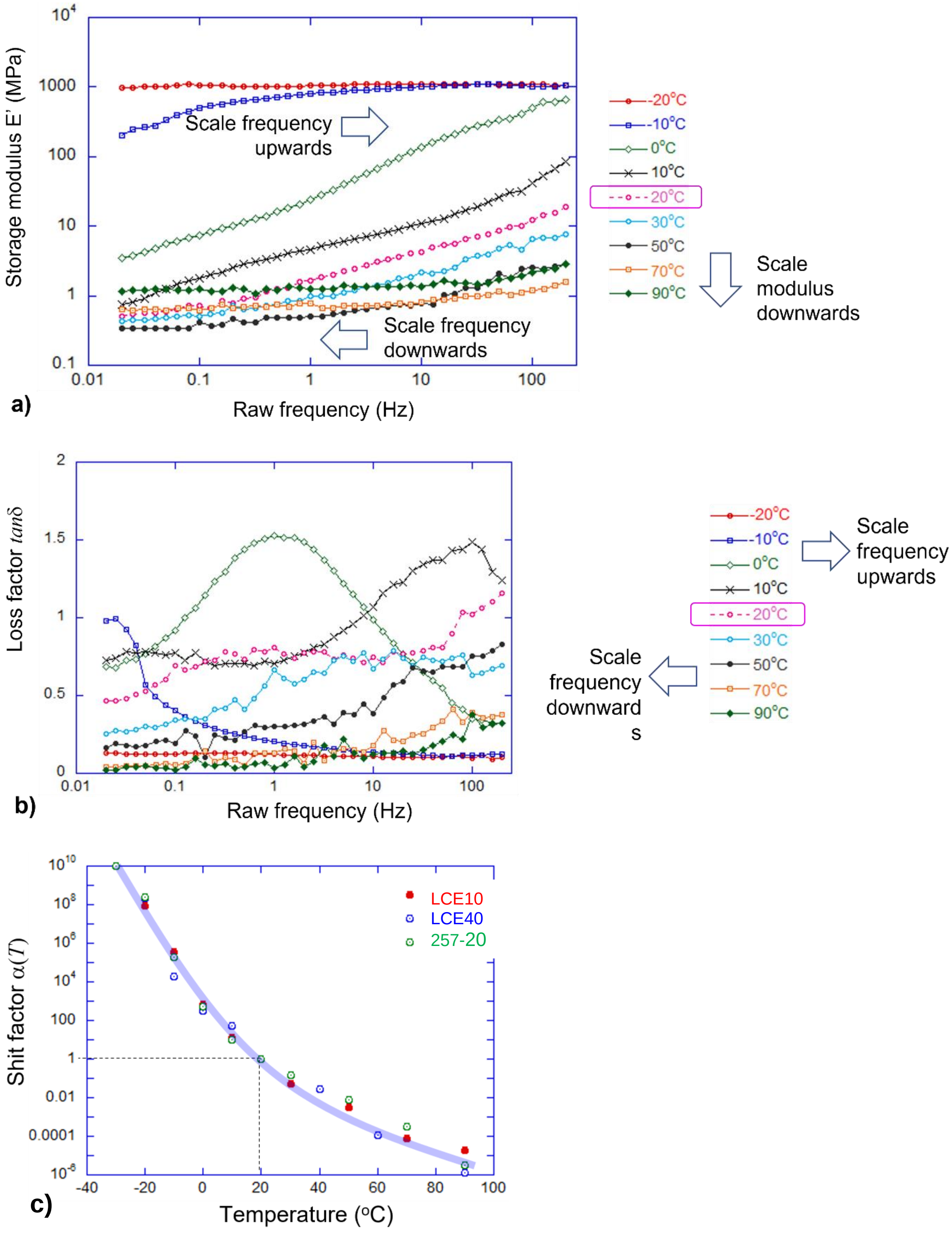

Fig. S2. Illustration of the time-temperature superposition for LCE materials, with the reference temperature of 20°C in the nematic phase. (a) Superposition of the storage modulus, (b) superposition of *tan*δ, (c) the shift factors for different LCE materials, with the reference T=20°C. The 'fitted' line is a guide to an eye: the classical WLF expression for α(T) does not apply here, since we are not dealing with the plain glass transition. Nevertheless, the superposition principle applies well, and remarkably – the empirically obtained shift factors are apparently the same for LCE materials with different crosslinking densities.

3. Hopkinson bar experiments

The standard split-Hopkinson bar rig is composed of three axially aligned rods: the striker, incident, and transmission bars, see Figure S2. The specimen is sandwiched between the incident and transmission bars in a stress-free state prior to the start of the experiment. The test is actuated when the striker bar is accelerated using compressed gas from a gun barrel. When the striker bar impacts the end of the incident bar, an incident stress wave is generated that propagates along the bar until it reaches the sample. The specimen is compressed at a high deformation rate, although very low strain amplitude (measured in a few μm) when the incident wave arrives at the end of the incident bar. Part of the wave is transmitted through the damping sample into the transmission bar, and part is reflected in the incident bar as a reflected wave. Strain gages mounted on the incident and transmission bars allow measurement of the specimen nominal stress, strain, and strain rate. Pulse shaping, and the specimen shaping, area critical step in achieving a constant nominal strain rate deformation and equilibrated stress in the sample, both of which are required for a valid experiment. Small disks of annealed copper, or "pulse shapers" are placed on the impact end of the incident bar to shape the pulse.

When examining soft elastomeric samples, it is helpful to minimize the modulus (impedance) mismatch between the incident bar and the sample (and the same between the transmitted bar and the sample), which is achieved by using soft magnesium alloy instead of steel. The sketches and photos in Fig. S3 illustrate the flat-face impact of the incident Hopkinson bar. In the main text, we show a similar test when a steel ball is fired into the target instead of the incident bar. The target (transmitted) bar, and the strain gauge readout are the same in this test, while the impact and rebound energies are measured by recording the ball speed in both directions.

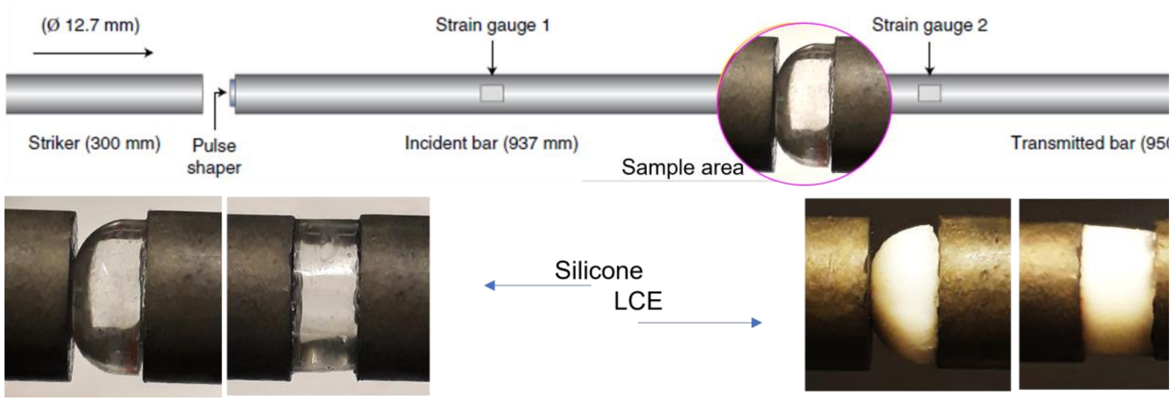

Fig. S3. Schematic of the design. The striker bar generates a compression wave in the incident bar, the shape of which is recorded by the strain gauge 1, first in passing towards the impact, and then in reflection. After the impact, the transmitted mechanical energy forms the compression wave travelling along the target (transmitted) bar, which is recorded by the strain gauge 2.

We have not shown the results of the flat impact in the main paper, but here this is illustrated in Fig. S4, where a flat-face cylindrical elastomer pad is impacted by the Hopkinson bar. Monitoring the power transmitted into the target bar, and reflected back into the incident bar, we can monitor the full energy budget of impact. It is curious that almost none of the impact energy is transmitted through the elastic PDMS: only the reflection is noticeable – in contrast to much slower reaction of both LCEs and a small fraction of energy transmitting through (not the great difference in scales on two axes in Fig. S4). However, importantly, all three materials show exactly the same fraction of energy dissipated, which is because in this flat-impact

geometry only the plane compressional wave is initiated, and no shear component is present to 'help' with internal dissipation.

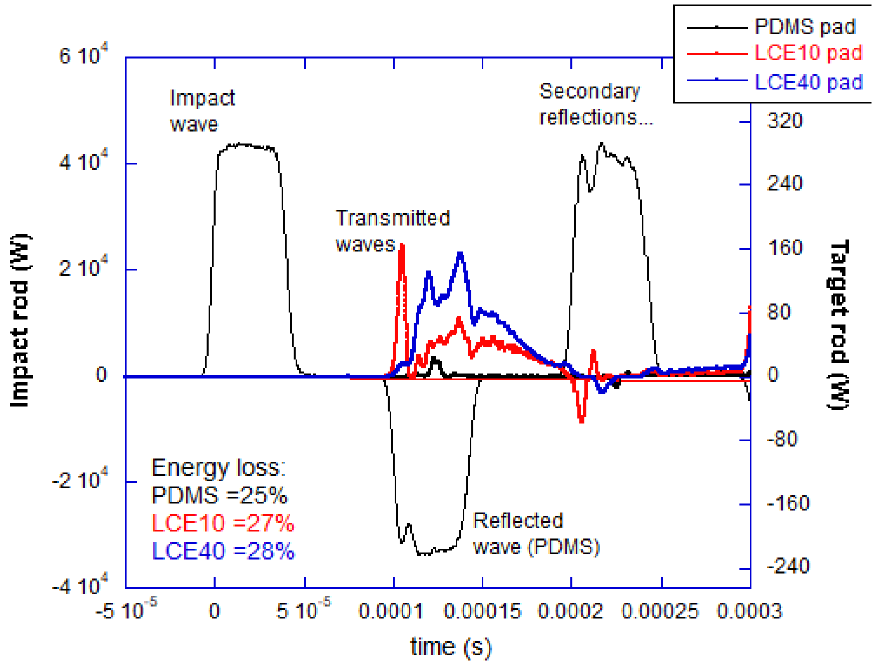

Fig. S4. The split Hopkinson bar impact on a flat-face cylinder elastomer pad. The impact energy is 2J, with 25-28% of impact energy absorbed in the elastomers.

It is important to establish a correspondence between the impact measurements, providing data in real time (cf. Figure 3 in the main text), and the frequency domain where we see both the material properties (such as Master Curves in Figure 2 in the main text) and the elastic waves. For this, we could Fourier transform the impact data. As an example, working with power transmission in the ball impact (Figure 3a), the spectral distribution of the response is shown in Fig. S5. As expected for the sharp, 0.2 ms pulse in real time, its frequency distribution is a relatively flat-top until the cutoff frequency – the logarithmic frequency scale in Fig. S5 over-emphasizes this point, and marks the 1/10 amplitude cutoff for the three samples. We find for PDMA this cutoff at ca.7.5 kHz, for LCE40 at ca. 10.6 kHz, and for LCE10 at ca. 10.2 kHz (very approximately, given the FT error and the arbitrary choice of 1/10th amplitude cutoff).

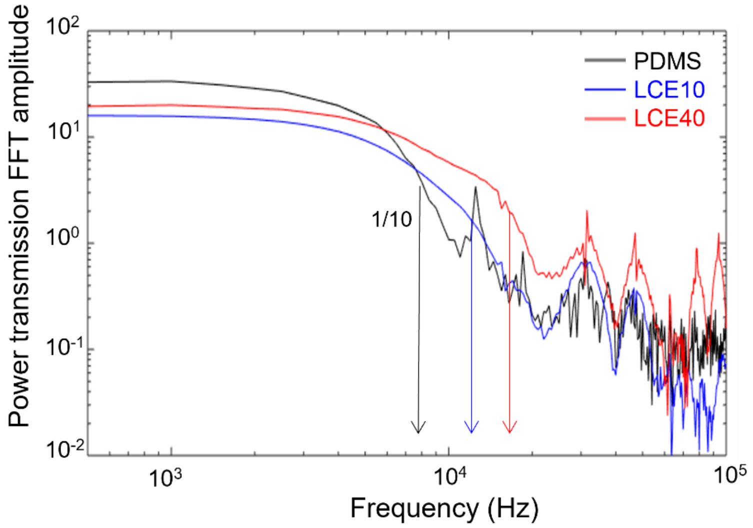

Fig. S5. The FFT of impact traces of Figure 3(a) in the main text.

We can now turn to the Master Curves for *tan*δ(ω), such as in Figure 2(b) in the main text, and use the simple analytic relationship between *tan*δ and the fraction of energy loss:

$$\eta = 1 - \frac{2 + \tan\delta(\omega)(2\delta(\omega) - \pi)}{2 + \tan\delta(\omega)(2\delta(\omega) + \pi)}$$

(which is obtained by simple integration over relevant sections of the Lissajous plot of the phase-shifted stress and strain). This gives the fraction of energy lost at each frequency and, given the Master Curves for *tan*δ(ω), one could integrate this up to the cutoff frequency determined above for each material. This clearly captures the significant portion of the enhanced *tan*δ region in the Master Curves and so predict the high dissipation fraction (as we observed by integrating the measured power over the time domain of the pulse). Hence the qualitative correspondence is strong. However, at this time we are unable to make the quantitative correspondence, because directly integrating $\int \eta(\omega) d\omega$ gives a value of 'wrong' dimensionality, and a more involved theoretical analysis is required to obtain the matching quantity. This is something we are interested in doing in the future.

4. Excitation and transmission of elastic waves

The LCE samples were prepared in a cylindrical shape of a diameter of 17 mm and a length of 15-16 mm. The samples were dynamically measured using a laser vibrometer set up, as shown in Fig. S6. The setup was comprised of a Modal Shop K2007E01 dynamic shaker, a PCB 288D01 impedance head and a Polytec PDV 100 laser vibrometer. The laser vibrometer measures the surface velocity of the flat top surface of the samples and has a frequency measurement range from 0.5 Hz up to 22 kHz with precise linearity throughout the frequency range. It uses a Helium-Neon class 2 laser (output power less than 1 mW) and a wavelength of 623 nm. A BNC cable was used to link the laser vibrometer to a Polytec junction box which transfers the measurements to a processing software (Polytec VibSoft). An internal trigger was set up to the time of sending and receiving of the signals. A periodic chirp excitation was sent through the junction box to the dynamic shaker which can handle a stroke force of up to 13 mm (far beyond the maximum stroke length that occurred during our measurements).

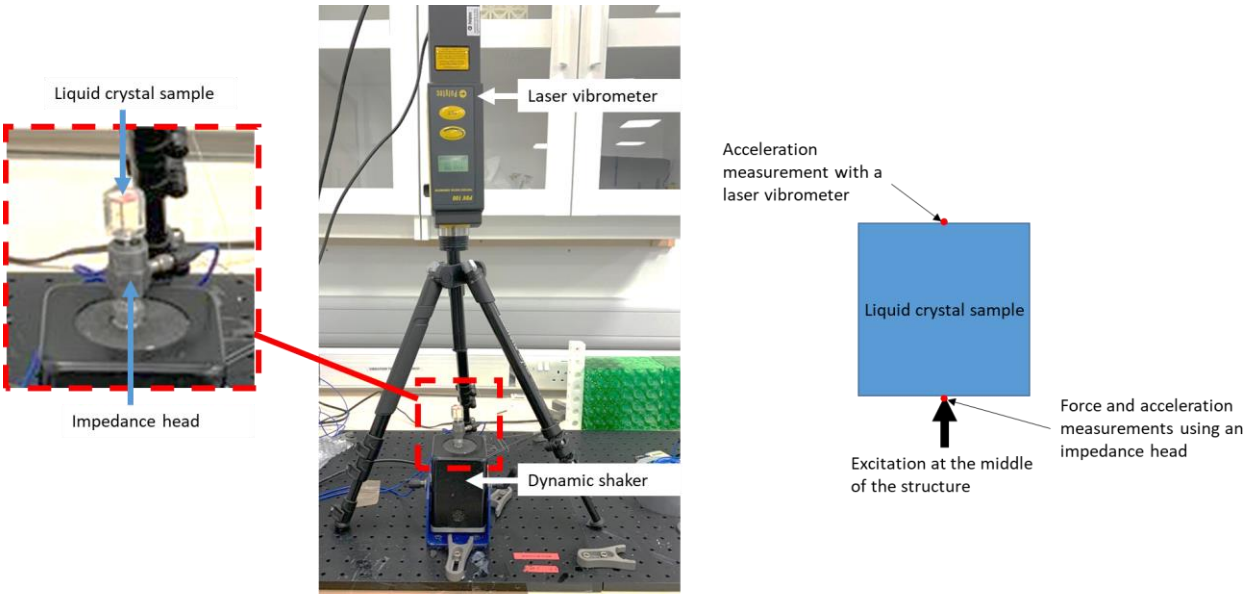

Fig. S6. Illustration of the vibrometer setup, with the acoustic transducer at the bottom of the cylindrical sample, and the laser range meter at the top.

The output power of the shaker was 55 W and it used natural air cooling. Sufficient cooling time was given after measurement of each liquid crystal sample to ensure the dynamic shaker was in full working order. The PCB 288D01 is attached to the output end of the dynamic shaker to measure the force output by the shaker at each frequency, which is equal to the force input to the bottom of the liquid crystal sample. The impedance head has a sensitivity of 22.4 mV/N, a force measurement range of up to 222 N and a frequency measurement range from 0.7 Hz to 7000 Hz. The LCE sample was glued to the impedance head. A reflective paper was attached to the top of the LCE cylinder to increase the measurement efficiency of the laser vibrometer which is shined on the reflective paper.

A digital twin of the LCE sample was modelled in CAD. The model was then imported into a commercial finite element analyser and was modelled with the properties of a typical elastomer (density of 1200 kg/m^3, Poisson's ratio ~ 0.5 and Bulk modulus of 16.7 MPa). The modal analysis describes the vibration behaviour of the samples at a certain frequency region. This is essential to understand the primary direction of vibration modes at the resonances that we get from the experiment. The calculations were done using free-free boundary conditions. Hex-dominant elements were used to mesh the model and convergence of the results was ensured. It was shown that the primary longitudinal mode of the liquid crystal occurs at around 836 Hz as shown in Fig. S7. Other modes with longitudinal and torsional movements exist in frequency regions close to that of the main longitudinal mode shape. However, depending on the nature of the force exerted on the sample, a mode shape will be excited. Since our experiment included longitudinal excitation, then the mode shapes associated with the torsional motion (1144 Hz) and other transverse motions do not manifest themselves in the transmissibility graphs.

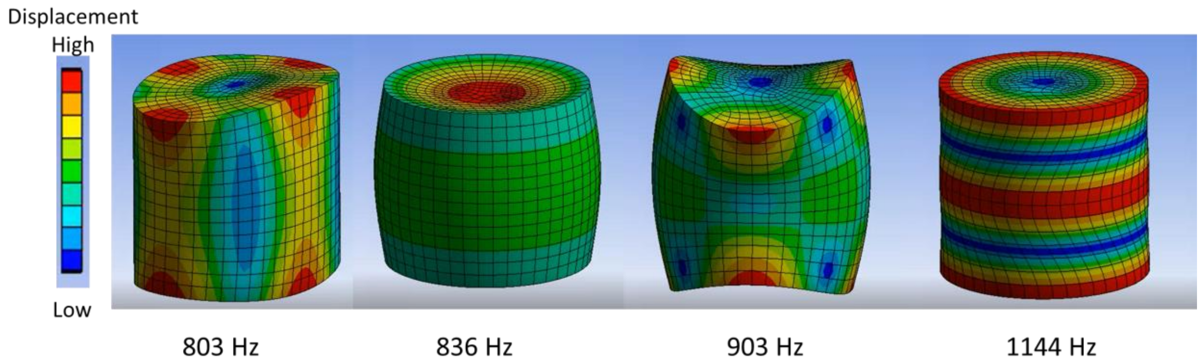

Fig. S7. Mode shape analyses of a typical liquid crystal cylinder.
